# Supplementary material for: FastqPuri: high-performance preprocessing of RNA-seq data
Source: BMC Bioinformatics. 2019 May 3;20:226. doi: 10.1186/s12859-019-2799-0 (PMC6500068; doi:10.1186/s12859-019-2799-0)
Supplement: Supplementary file 2 — Archive of FastqPuri. Archive containing all files needed to install and run FastqPuri v1.0.6. Date stamp March 22, 2019. (GZ 47,819 kb) [file 12859_2019_2799_MOESM2_ESM.gz › FastqPuri-1.0.6/html/tree_8h_source.html]

FastqPuri: include/tree.h Source File


FastqPuri


- include

tree.h

Go to the documentation of this file.

1 /\*\*\*\*\*\*\*\*\*\*\*\*\*\*\*\*\*\*\*\*\*\*\*\*\*\*\*\*\*\*\*\*\*\*\*\*\*\*\*\*\*\*\*\*\*\*\*\*\*\*\*\*\*\*\*\*\*\*\*\*\*\*\*\*\*\*\*\*\*\*\*\*\*\*\*\*

2  \* Copyright (C) 2017 by Paula Perez Rubio \*

3  \* \*

4  \* This file is part of FastqPuri. \*

5  \* \*

6  \* FastqPuri is free software: you can redistribute it and/or modify \*

7  \* it under the terms of the GNU General Public License as \*

8  \* published by the Free Software Foundation, either version 3 of the \*

9  \* License, or (at your option) any later version. \*

10  \* \*

11  \* FastqPuri is distributed in the hope that it will be useful, \*

12  \* but WITHOUT ANY WARRANTY; without even the implied warranty of \*

13  \* MERCHANTABILITY or FITNESS FOR A PARTICULAR PURPOSE. See the \*

14  \* GNU General Public License for more details. \*

15  \* \*

16  \* You should have received a copy of the GNU General Public License \*

17  \* along with FastqPuri. \*

18  \* If not, see <http://www.gnu.org/licenses/>. \*

19  \*\*\*\*\*\*\*\*\*\*\*\*\*\*\*\*\*\*\*\*\*\*\*\*\*\*\*\*\*\*\*\*\*\*\*\*\*\*\*\*\*\*\*\*\*\*\*\*\*\*\*\*\*\*\*\*\*\*\*\*\*\*\*\*\*\*\*\*\*\*\*\*\*\*\*\*/

20

30 #ifndef TREE\_H\_

31 #define TREE\_H\_

32 #include <stdint.h>

33 #include "defines.h"

34 #include "fa\_read.h"

35

40 typedef struct \_node {

41  struct \_node \*children[T\_ACGT];

42 } Node;

43

60 typedef struct \_tree {

61  uint32\_t L;

62  uint32\_t pool\_count;

63  uint32\_t pool\_available;

64  uint32\_t nnodes;

65  Node \*\*pool\_2D;

66 } Tree;

67

68 Node \*get\_new\_pool(Tree \*tree\_ptr);

69

70 Node \*new\_node\_buf(Tree \*tree\_ptr);

71

72 void free\_all\_nodes(Tree \*tree\_ptr);

73

74 void insert\_Lmer(Tree \*tree\_ptr, char \*Lmer);

75

76 void insert\_entry(Tree \*tree\_ptr, Fa\_entry \*entry);

77

78 double check\_path(Tree \*tree\_ptr, char \*read, int Lread);

79

80 Tree \*tree\_from\_fasta(Fa\_data \*fasta, int L);

81

82 void save\_tree(Tree \*tree\_ptr, char \* filename);

83

84 Tree \*read\_tree(char \*filename);

85

86 /\* static functions

87  \* check\_path(Tree \*tree\_ptr, char \*Lmer, int Lread);

88  \* \*/

89

90 #endif // endif TREE\_H\_

check\_path

double check\_path(Tree \*tree\_ptr, char \*read, int Lread)

checks if read is found in tree and outputs a score

**Definition:** tree.c:199

insert\_Lmer

void insert\_Lmer(Tree \*tree\_ptr, char \*Lmer)

Lmer insertion in the tree (depth L).

**Definition:** tree.c:145

\_fa\_data

stores sequences of a fasta file

**Definition:** fa\_read.h:46

fa\_read.h

reads in and stores fasta files

Node

struct \_node Node

Node structure: formed out of T\_ACGT pointers to Node structure.

free\_all\_nodes

void free\_all\_nodes(Tree \*tree\_ptr)

frees the whole tree structure

**Definition:** tree.c:117

\_node

Node structure: formed out of T\_ACGT pointers to Node structure.

**Definition:** tree.h:40

read\_tree

Tree \* read\_tree(char \*filename)

read tree from file

**Definition:** tree.c:309

get\_new\_pool

Node \* get\_new\_pool(Tree \*tree\_ptr)

reallocs pool\_2D (++NPOOL\_2D) if all existing nodes have been used

**Definition:** tree.c:44

\_tree::pool\_available

uint32\_t pool\_available

**Definition:** tree.h:63

\_node::children

struct \_node \* children[T\_ACGT]

**Definition:** tree.h:41

tree\_from\_fasta

Tree \* tree\_from\_fasta(Fa\_data \*fasta, int L)

create Tree structure from fasta structure.

**Definition:** tree.c:177

defines.h

Macro definitions.

save\_tree

void save\_tree(Tree \*tree\_ptr, char \*filename)

saves Tree to disk in filename

**Definition:** tree.c:237

insert\_entry

void insert\_entry(Tree \*tree\_ptr, Fa\_entry \*entry)

fasta entry insertion in the tree (depth L).

**Definition:** tree.c:162

\_tree::pool\_count

uint32\_t pool\_count

**Definition:** tree.h:62

Tree

struct \_tree Tree

structure containing a T\_ACGT-tree.

\_tree::nnodes

uint32\_t nnodes

**Definition:** tree.h:64

new\_node\_buf

Node \* new\_node\_buf(Tree \*tree\_ptr)

moves to the next node (allocating new memory if necessary)

**Definition:** tree.c:84

\_tree::pool\_2D

Node \*\* pool\_2D

**Definition:** tree.h:65

\_tree

structure containing a T\_ACGT-tree.

**Definition:** tree.h:60

\_fa\_entry

fasta entry

**Definition:** fa\_read.h:38

\_tree::L

uint32\_t L

**Definition:** tree.h:61

T\_ACGT

#define T\_ACGT

**Definition:** defines.h:84


---

Generated on Mon Mar 19 2018 23:42:01 for FastqPuri by  

 1.8.14
